# Glucocorticoid Induced Leucine Zipper in Lipopolysaccharide Induced Neuroinflammation

**DOI:** 10.3389/fnagi.2018.00432

**Published:** 2019-01-25

**Authors:** Emily Witek, Debra Hickman, Debomoy K. Lahiri, Mythily Srinivasan

**Affiliations:** ^1^Laboratory Animal Resources and Center, Department of Internal Medicine, Indiana University School of Medicine, Indianapolis, IN, United States; ^2^Department of Psychiatry, Stark Neuroscience Research Institute, Indiana University School of Medicine, Indianapolis, IN, United States; ^3^Department of Oral Pathology, Medicine and Radiology, Indiana University School of Dentistry and Indiana University—Purdue University Indianapolis, Indiana University School of Medicine, Indianapolis, IN, United States

**Keywords:** neuroinflammation, glucocorticoid induced leucine zipper, Alzheimer’s disease, toll like receptor-4, cytokines

## Abstract

Glucocorticoids (GCs) are steroid hormones secreted as the end-product of the neuroendocrine stress cascade. Both absence and elevated GC mediate neurotoxic responses, suggesting that a narrow window ranging from physiological to slightly high GC mediate protective responses. The beneficial effects of GC are attributed to the transactivation of regulatory proteins and inhibition mediated by glucocorticoid receptor (GR) interactions with other co-factors. The glucocorticoid induced leucine zipper (GILZ) is a gene strongly upregulated by GC and mediates many of the anti-inflammatory and anti-proliferative effects of GC. Although GILZ is constitutively expressed in many tissues including the brain, the expression has been shown to occur with varying dynamics suggesting that the local milieu modulates its expression with consequent effects on cellular responses. Here we investigated the expression profile of GILZ in lipopolysaccharide (LPS) mediated neuroinflammation model of Alzheimer’s disease (AD). Our data suggest that the GILZ expression is downregulated in neuroinflammation correlating inversely with the pro-inflammatory cytokines and innate immune responses.

## Introduction

Increased plasma glucocorticoids (GCs) are features of both aging and Alzheimer’s disease (AD; Huang et al., 2009). Endogenous GC released by the adrenal glands play a key role in the homeostasis by regulating energy metabolism, coordinating immune responses and orchestrating the adaptive responses to stress. Increased secretion of GC can occur in response to various stimuli such as low plasma levels of GC, psychological or physical stress. The elevated GC in general is suppressed by a negative feedback loop, mainly regulated by the hypothalamic pituitary adrenal axis (HPA; Abrahám et al., 2006; Vyas et al., 2016). However, aging has been associated with a decreased sensitivity of the HPA axis to GC feedback, which in turn leads to prolonged elevated levels of plasma GC (Lupien et al., 1999).

Although the profound anti-inflammatory effects of GC suggest a neuroprotective role, emerging evidence strongly implicate elevated GC in potentiating the pro-inflammatory responses and neuroinflammation in the central nervous system (CNS). Multiple mechanisms are suggested for the effects of high GC on brain including dysregulated HPA axis, alterations in glial functions as well as structural remodeling of neurons with synaptic loss (Duque Ede and Munhoz, 2016; Vyas et al., 2016). Classically, GC act by binding the glucocorticoid receptor (GR) or the mineralocorticoid receptor (MR) in the cytoplasm. The ligand bound GR complex translocate to the nucleus and mediate cellular responses by genomic mechanisms via specific glucocorticoid response elements (GREs) in the DNA or by non-genomic protein: protein interactions. The therapeutic efficacy of GC are attributed to the combined effects of transactivation of regulatory proteins and inhibition of the pro-inflammatory transcription factor, the nuclear factor-kappa B (NF-κB) by tethering (Newton, 2000; Srinivasan and Lahiri, 2017).

Glucocorticoid induced leucine zipper (GILZ) is a member of the TSC22 family of proteins that share the TSC box and the leucine zipper domains. It was originally identified as a gene rapidly induced by dexamethasone in lymphocytes (D’Adamio et al., 1997). More recently, its constitutive expression is reported in multiple cell types such as the microglia, epithelial cells, skeletal muscle cells, osteocytes and cardiomyocytes (Shi et al., 2003; Yachi et al., 2007; Liu et al., 2013; Pan et al., 2014). Functionally, GILZ mimics the anti-inflammatory and the anti-proliferative effects of GC by inhibiting the activated NF-κB and by upregulating the transcription of anti-inflammatory genes such as IL-10 (Berrebi et al., 2003; Mazzon et al., 2014).

GILZ is induced in the brain by both MR and GR mediated transactivation (Bergann et al., 2011). Since GC exhibit over 10-fold higher affinity for MR than GR, it is likely that under physiological conditions of low GC, GILZ induced by the high affinity MR assists in maintaining the homeostasis in the CNS. When GC is elevated, impaired feedback and increased cytoplasmic retention of GC could result in reduced GILZ induction contributing to dysregulated cellular responses (Rupprecht et al., 1993; Ogita et al., 2012). Stress induced GC upregulates the toll like receptors (TLRs) in microglia primed by immune activation (Frank et al., 2010). In addition, GILZ induction has been shown to occur with varying dynamics in different tissues *in vivo*, suggesting that the local environment can modulate its expression with potential effects on cellular responses (Ayyar et al., 2015).

In this study, we investigated the role of GILZ in lipopolysaccharide (LPS) induced neuro-inflammation and its relation to the innate immune response in the brain. LPS induced inflammation is a widely used model to study the mechanisms of neuroinflammation preceding neurodegeneration in AD pathogenesis (Nazem et al., 2015). Our data suggest that the GILZ is downregulated and inversely related to the increased TLR-4 expression in the brain in LPS induced neuroinflammation.

## Materials and Methods

### Animals and Disease Induction

LPS from Escherichia coli 026:B6, = 10,000 EU/mg, was purchased from Sigma (Sigma Aldrich, St. Louis, MO, USA). Groups of adult C57/6J mice (five/group) were administered intraperitoneally either LPS at 250 mg/kg in 100 μl of sterile saline followed by corn oil daily for 6 days (Ifuku et al., 2012; Catorce and Gevorkian, 2016) or only saline (100 μl) injections. Un-manipulated naïve mice were included as additional controls. The animal experiments were approved by the Institutional animal care and use committee of the Indiana University Purdue University at Indianapolis.

### Histology and Immunohistochemistry

The mice were sacrificed on day 7 by intra-cardial perfusion of 25 ml of 0.9% saline. Blood was collected prior to infusion. Brain from each mouse was removed, one half was immediately frozen and the other half fixed in 4% paraformaldehyde for paraffin embedding. Hematoxylin and eosin stained sections were assessed for inflammation. Serial 10 μ thick coronal sections were immunostained for markers of microglial cells and astrocytes, GILZ and NF-κB p65 (Ray et al., 2009). Briefly, after deparaffinization, hydration, antigen retrieval and blocking non-specific binding (Enzo biosciences, Farmingdale, NY, USA) the sections were incubated overnight with the anti-Iba-1 (Clone:AIF1) or anti-glial fibrillary acidic protein (GFAP; clone GA5) or anti-NFκB p65 (clone 12H11; EMD Millipore Corporation, Temecula, CA, USA) or anti-GILZ polyclonal antibody (Cat #PA5-34506; Invitrogen, Carlsbad, CA, USA) primary antibody. Brown staining observed by using the IHC select peroxidase conjugated streptavidin-biotin system was considered positive (Millipore). For each marker, the specificity of staining was confirmed by incubating a separate set of sections with secondary antibodies alone. The area of positive staining in the dentate gyrus (DG) and the *Cornu Ammonis* (CA1 and CA3) regions of each stained section was quantified by ImageJ software (NIH Image 1.62).

### Enzyme Linked Immunosorbent Assay

The cytokines IL-6, TNF-α, and IL-17 in serum from each blood sample was quantitated using OptEIA kits (BD Biosciences, San Jose, CA, USA).

### Real Time Polymerase Chain Reaction

Total cellular RNA isolated from each brain tissue using Qiagen kit (Invitrogen, Carlsbad, CA, USA) was reverse transcribed using iScript cDNA kit (Biorad, Hercules, CA, USA). Equal amount of cDNA was used for amplification of β-actin, IL-1β, IL-12, CD14, TLR-4 and CD4 by quantitative real time polymerase chain reaction (PCR) using SYBR green/ROX qPCR master mix (SA Biosciences, Frederick, MD, USA) on the ABI Prism 7000 sequence detection system (Applied Biosystem, Foster City, CA, USA; Srinivasan and Janardhanam, 2011). The primers (gene accession number) used were: β-actin (NM_007393): F-5′TCATGAAGTGTGACGTTGACATCCGTA3′; R-5′CCTAGAAGCATTTGCGCTGCACGATGG3′ (286bp); IL1β (NM_008361) F-5′AGCTGATGGCCCTAAACAGA3′; R-5′GGTCGGAGATTCGTAGCTGG3′ (89bp); CD14 (NM_009841) F-5′GAGCTAGACGAGGAAAGTTGT3′; R-5′ACCGTAAGCCGCTTTAAGGACAGA3′ (206bp); GILZ (NM_001077364) F-5′CTAGCTCCGCAGGTGCGCAC3′; R-5′CGAGGCCAACAGGTGAGCGG3′ (122bp), IL-12 (NM_001303244) F-5′GGAAGCACGGCAGCAGAATA3′; R-5′AACTTGAGGGAGAAGTAGGAATGG3′ (179bp), TLR4 (NM_021297) F-5′CAGTCGGTCAGCAAACGCCTTCTTC; R-5′TGTAACTGGTGGCAGCGCA3′ (216bp); CD4 (NM_013488) F-5′GAGAGTCAGCGGAGTTCTC3′; R-5′CTCACAGGTCAAAGTATTGTTG3′ (182bp). The gene specific threshold cycle (Ct) was corrected by subtracting the Ct for the housekeeping gene β-actin. The magnitude of change in each gene was determined by the 2^−ΔCt^ method. Each measurement of a sample was performed in duplicates and the experiments were repeated at least two times.

### Statistical Analysis

Pairwise Student’s *t*-test was used to determine statistical significance in the cytokine and mRNA expression between the naïve, saline and LPS treated groups. *p* < 0.05 was considered significant. The relationship between the GILZ and TLR4 transcripts was evaluated by regression analysis, Pearson and Spearman correlation coefficient.

## Results

### LPS Induced Neuroinflammation Exhibits Increased Gliosis and Reduced GILZ Protein Expression

**Figure 1:**
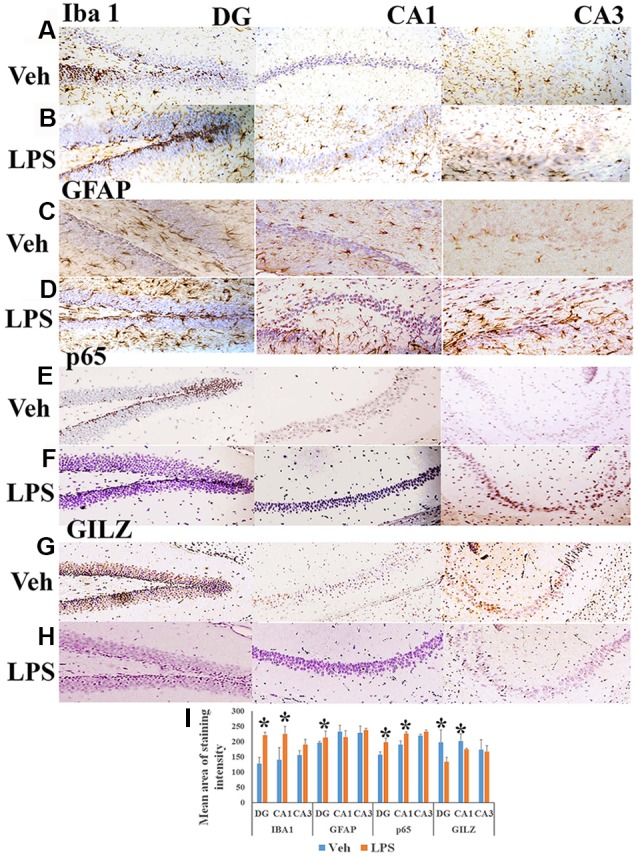
Immunohistochemistry of hippocampus of mouse induced neuroinflammation. Shows representative IHC section stained for Iba+ microglia **(A,B)**, glial fibrillary acidic protein (GFAP)+ astrocytes **(C,D)**, nuclear factor-kappa B (NF-κB) p65+ **(E,F)** and GLIZ+ cells **(G,H)** in the hippocampus of mouse subjected to lipopolysaccharide (LPS) induced neuroinflammation and vehicle treated mouse as indicated. Panel **(I)** shows the mean staining area of the 3,3′-diaminobenzidine (DAB)-positive cells depicting microglia, astrocytes, NF-κB p65 or GILZ+ cells in groups of mice induced neuroinflammation or vehicle treated mice (DG, dentate gyrus; CA 1, Cornu Ammonis 1; and CA 3, Cornu Ammonis 3; representing specific regions of the hippocampus). *p < 0.05 as compared with vehicle treated mice.

Peripherally administered LPS has been shown to increase the number of activated microglial cells as well as astrogliosis in the brain (Qin et al., 2007; Catorce and Gevorkian, 2016). We observed that the mean area of Iba+ microglial (Figures 1B,I) and GFAP+ astrocytes staining (Figures 1D,I) was higher in specific regions of the hippocampus of mice subjected to LPS induced neuroinflammation as compared to that in vehicle treated mice (Figures 1A,C,I). We also observed that the p65 immununostaining was higher in the DG and CA1 regions of the hippocampus of LPS administered mice (Figures 1F,I) as compared to that of the control mice (Figures 1E,I). While the control group of mice exhibited increased GILZ staining (Figures 1G,I), GILZ+ cells were fewer in DG and CA1 region the hippocampus of mice subjected to LPS induced neuroinflammation (Figures 1H,I).

### The GILZ Transcript Correlates Inversely With the Innate Immune and Inflammatory Transcripts in LPS Induced Neuroinflammation

**Figure 2:**
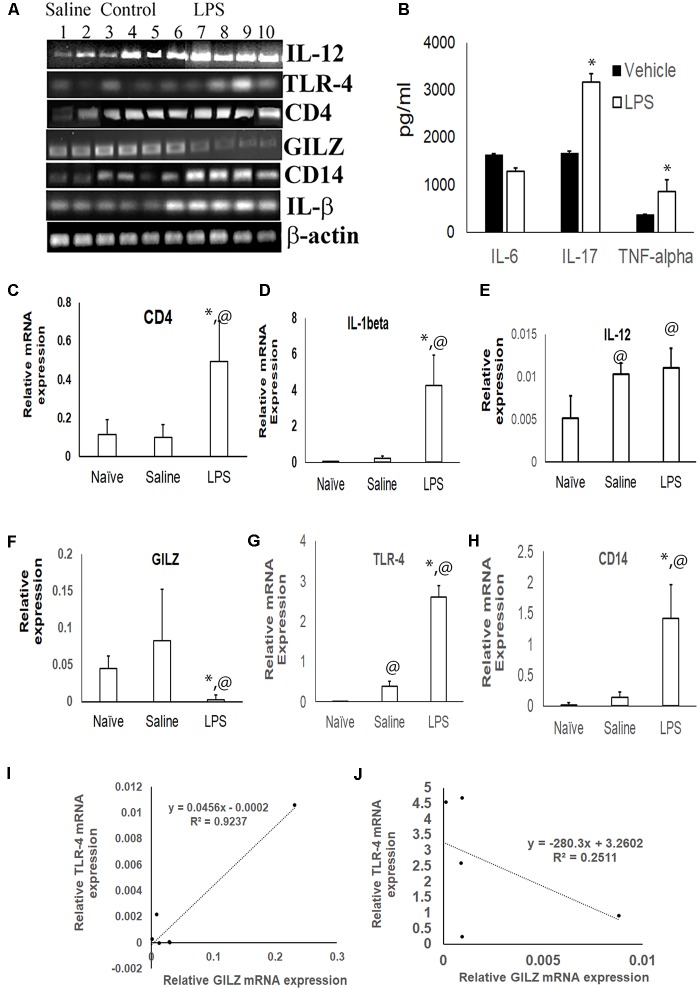
Effect of LPS induced neuroinflammation in the expression profiles of inflammatory markers and GILZ: groups of adult C57BL/6 mice were subjected to LPS induced neuroinflammation as described in the “Materials and Methods” section. Equal quantity of cDNA isolated from brain tissues were amplified for IL-1β, CD14, toll like receptor-4 (TLR-4), IL-12, CD4 and β-actin mRNA by quantitative real time polymerase chain reaction (PCR). **(A)** Gel electrophoresis of the PCR products β-actin IL-1β, CD14, TLR-4, GILZ, IL-12 and CD4. Panel **(B)** shows indicated inflammatory cytokines in the circulation as determined by enzyme linked immunosorbent assay (ELISA). **(C–H)** Relative mRNA quantitation of the indicated product with respect to that of housekeeping gene β-actin is shown. Data are average ± SD. * and ^@^*p* < 0.05 with respect to the vehicle treated group or naïve group respectively. Panel **(I,J)** shows data from regression analysis for GILZ and TLR4 transcripts in mice administered saline **(I)** or LPS **(J)**.

The inflammatory cytokines TNF-α and IL-17 was significantly higher in the serum of mice subjected to LPS induced neuroinflammation as compared to the serum of control group (Figure 2B). TLR4 is one of the main LPS recognizing receptors that respond to inflammatory stimuli and mediate NF-κB signaling pathway in microglial cells (Yu et al., 2015; Lykhmus et al., 2016). We observed that the expression of the TLR4 mRNA (Figures 2A,G) and that of its co-receptor CD14 (Figures 2A,H) was upregulated in the brain tissues of mice subjected to LPS induced neuroinflammation as compared to that in control mice. The upregulation was also observed for the transcripts of inflammatory markers CD4 and IL-1α (Figures 2A,C–E). In contrast, the GILZ mRNA was significantly lower in the brain tissues of mice subjected to LPS induced neuroinflammation as compared to that of the control group (Figures 2A,F). Regression analysis suggests a strong positive correlation between GILZ and TLR-4 (Spearman *R* = 0.9) expression in individual mouse in the control group (Figure 2I). This relationship was lost in individual mice subjected to LPS induced neuro-inflammation (Figure 2J).

## Discussion

Brain is well recognized as a major neural target for the actions of GC. The paradoxical effects of GC in neuronal survival and death have been attributed to the concentration and duration of exposure of GC and the bioavailability of receptors. In physiological conditions and during circadian trough, GC preferably bind high-affinity MR and mediate neuroprotective responses. High GC on the other hand promote neuronal injury by increasing inflammation and potentiating other cytotoxic mechanisms (Duque Ede and Munhoz, 2016; Kalafatakis et al., 2016). We postulated that since GILZ is an early and rapidly induced gene by GC, it is likely that it influences these processes.

Intraperitoneal LPS upregulate inflammatory cytokines which in turn increase glucocorticoid secretion. Yet, considerable evidence suggest that the LPS induced neuroinflammation is aggravated by higher circulating GC or chronic stress induced GC or exogenous GC due to development of glucocorticoid resistance and impaired feedback (Weidenfeld and Yirmiya, 1996; Pérez-Nievas et al., 2010; Vyas et al., 2016). We observed that the GILZ expression was reduced in the hippocampus in this model correlating negatively with the increased inflammatory markers. Others have reported reduced GILZ expression in LPS induced uveitis (Gu et al., 2017). Previously, reduced GILZ expression and elevated inflammatory cytokines in circulating lymphocytes were correlated with lower hippocampal volume in patients with major depressive disorder (Frodl et al., 2012). In contrast, water immersion stress that enhance endogenous GC was shown to upregulate neuronal GILZ in the mouse hippocampus (Yachi et al., 2007). The discrepancy could be attributed to the nature of the stimuli and the method used, *in situ* hybridization vs. quantitative PCR (this study). Pertinently, GILZ has been suggested to exert cell-context dependent functions ranging from inhibition of inflammation and immune responses to modulation of proliferation and cell death in multiple cell types (Cohen et al., 2006; Liu et al., 2013; Pan et al., 2014).

Data from serial gene expression analysis that characterized the global transcriptional effects of MR and GR signaling in GR deficient HT-22 neuronal cells showed that the GILZ is upregulated only in cells co-expressing MR and GR (Rozeboom, 2008). These observations suggest that the GILZ could contribute to the process of neuronal survival and apoptosis, perhaps in a GC concentration dependent manner. Our observations of higher GILZ expression in the brain of naïve mice suggest that in health GILZ is potentially upregulated by MR mediated transactivation. The reduced GILZ expression in LPS induced TLR-4 mediated inflammation perhaps reflects cytoplasmic GR retention with consequent reduced GILZ transactivation. Similar inverse correlation between the TLR-4 and GILZ expression is reported in alveolar macrophages (Hoppstadter et al., 2012).

Taken together, our data suggest a novel role for GILZ as a modulator of the neuroprotective and neuroinflammatory responses of GC. Further studies are underway to evaluate the effects of stress and inflammation in GILZ overexpressing mice. In addition, since GILZ sequesters activated NF-κB p65 in the cytoplasm and prevent inflammatory responses, it may represent a viable candidate for drug development (Ayroldi and Riccardi, 2009). Indeed, we have recently reported that analogs of the p65 binding motif of GILZ can inhibit amyloid beta induced inflammatory responses in mixed brain cells (Srinivasan et al., 2016).

## Author Contributions

MS, DH and DL were involved in concept development, data interpretation and manuscript preparation. EW and DH performed the animal experiments, data acquisition and interpretation.

## Conflict of Interest Statement

The authors declare that the research was conducted in the absence of any commercial or financial relationships that could be construed as a potential conflict of interest.
